# Matrix attachment regions as targets for retroviral integration

**DOI:** 10.1186/1743-422X-2-68

**Published:** 2005-08-19

**Authors:** Chassidy N Johnson, Laura S Levy

**Affiliations:** 1 Department of Microbiology & Immunology and Tulane Cancer Center, Tulane University School of Medicine, New Orleans, Louisiana, 70112, USA

## Abstract

**Background:**

The randomness of retroviral integration has been debated for many years. Recent evidence indicates that integration site selection is not random, and that it is influenced by both viral and cellular factors. To study the role of DNA structure in site selection, retroviral integration near matrix attachment regions (MARs) was analyzed for three different groups of retroviruses. The objective was to assess whether integration near MARs may be a factor for integration site selection.

**Results:**

Results indicated that MLV, SL3-3 MuLV, HIV-1 and HTLV-1 integrate preferentially near MARs, specifically within 2-kilobases (kb). In addition, a preferential position and orientation relative to the adjacent MAR was observed for each virus. Further analysis of SL3-3 MuLV insertions in common integration sites (CISs) demonstrated a higher frequency of integration near MARs and an orientation preference that was not observed for integrations outside CISs.

**Conclusion:**

These findings contribute to a growing body of evidence indicating that retroviral integration is not random, that MARs influence integration site selection for some retroviruses, and that integration near MARs may have a role in the insertional activation of oncogenes by gammaretroviruses.

## Background

An essential step in the replication cycle of all retroviruses is integration of the double-stranded DNA proviral form of the genome into host DNA. The degree of randomness of proviral integration has been debated for many years [1,2]. Studies have suggested that DNaseI hypersensitive sites [3-7], AT-rich regions [8], transcriptionally active regions [2,9-12], repeat elements including Alu and LINE elements [13] and regions of DNA bending, specifically regions with the most DNA distortion [14-18], are preferred sites of proviral integration. Alternatively, studies have shown that high levels of transcription disfavor integration of avian leukosis virus (ALV) [2]. The conflicting results that have been reported may be explained by the small sample sizes examined or by potential biases introduced from the cloning strategies used to identify insertion sites. In addition, many of the studies were performed *in vitro*, and thus did not take into account the native conformation of chromatin. Before the completion and publication of the human and mouse genome databases, theories for randomness of retroviral integration were difficult to prove or disprove because of the technical challenge of analyzing a large sample size of integrations from infected cells. Since publication of the genome databases, several studies have isolated and mapped hundreds of proviral insertion sites for murine leukemia virus (MLV), human immunodeficiency virus type-1 (HIV-1), avian sarcoma virus (ASV) and human T-cell leukemia virus type-1 (HTLV-1) [11,12,19,20]. For those viruses, the results showed preferential integration into transcriptionally active NCBI Reference Sequences (RefSeqs), but distinct patterns of integration were evident as well. These studies provided strong evidence that distinct viruses differ in proviral integration patterns, but that integration is clearly non-random. The specific pressures that influence site selection for retroviral integration remain incompletely understood.

Accumulating evidence indicates that retroviral integration site selection is influenced by properties of cellular DNA structure [11,21-24]. A recent large-scale study found that DNA structural features such as bendability and A-philicity served as preferred integration sites [22]. The present study was performed to assess the role of matrix attachment regions (MARs) in retroviral integration site selection. MARs are DNA sequences located at the bases of DNA loops that attach to the nuclear matrix, and are thus positioned near the machinery for DNA replication, transcription, RNA processing and transport (reviewed in [25]). There is no consensus sequence that defines a MAR; however, MARs are commonly found to have intrinsic DNA bending properties, to contain transcription factor binding sites, AT-rich stretches, sites for topoisomerase I and II binding and cleavage, and high unwinding potential [26,27]. MARs function as structural regulatory elements by organizing the DNA into loop domains. Studies have shown that MARs influence the expression of cellular genes, and can enhance viral gene expression when in the vicinity of viral promoters and enhancers [28-30]. This property has made the inclusion of MARs in gene therapy vectors attractive for enhanced and prolonged expression of the transgene in a specific cell-type or developmental stage [31-33]. MARs have been implicated in virus-mediated malignancies, particularly as targets of integration by small DNA tumor viruses. Specifically, integrated SV40, HBV, HPV16 and HPV18 have been found within or in close proximity to MARs in tumors or transformed cell lines [34]. Other reports indicate that HTLV-1 and HIV-1 may integrate preferentially near MARS [34,35].

The gammaretroviruses represent a group of mammalian oncogenic retroviruses typically associated with the induction of long-latency leukemia and lymphoma in the natural host. Gammaretroviruses do not encode an oncogene or any other gene to which their malignant potential can be directly attributed. Rather, their ability to induce tumors has been linked to a process termed insertional activation, in which integration of the proviral genome into host DNA is associated with activated expression of an adjacent oncogene. When the same genetic locus is observed to be interrupted by proviral integration in multiple independent tumors, it is inferred that the commonly interrupted locus encodes an oncogene whose activation is relevant to tumor induction [36-38]. Such a locus is referred to as a common insertion site (CIS). We recently described CISs utilized by a recombinant gammaretrovirus, MoFe2-MuLV (MoFe2), in T-cell lymphomas in the NIH/Swiss mouse. To construct MoFe2, the U3 region of the Moloney murine leukemia virus (M-MuLV) long terminal repeat (LTR) was substituted with homologous sequences from a natural isolate of feline leukemia virus termed FeLV-945 [39]. FeLV-945 is characterized by a unique motif in the U3 region of the LTR, which contains a single copy of the transcriptional enhancer followed downstream by the tandem triplication of a 21-bp sequence. Substitution of FeLV-945 LTR sequences into M-MuLV was shown to alter the pattern of insertional activation and to identify new CISs [40]. As described below, the identification of two potential MARs near a CIS in MoFe2-induced lymphomas suggested that MARs may represent a determinant of integration site selection. That hypothesis was addressed in the present study by analyzing the proximity of proviral integrations to MARs in lymphomas and in unselected cultured cells. The patterns of integration with respect to MARs were compared for three groups of retroviruses, including several murine gammaretroviruses, human deltaretrovirus (HTLV-1) and lentivirus (HIV-1).

## Results

**Figure 1:**
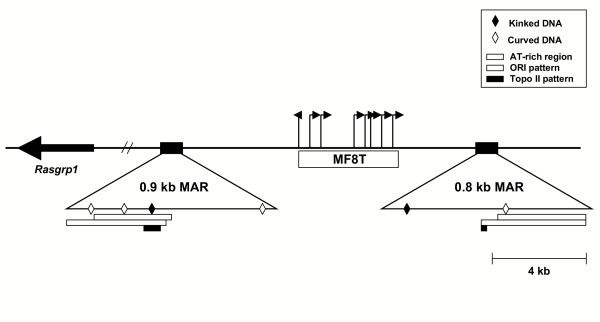
Physical map of the MF8T locus. Depicted is the 3.9-kb domain of common proviral insertion designated MF8T. Vertical lines represent the positions of the proviral integrations with the transcriptional orientation of provirus depicted by the direction of the arrow. Depicted is *Rasgrp1*, the predicted oncogene in the MF8T locus. Two predicted MARs of 0.9-kb and 0.8-kb in size are located 5.1-kb and 3.6-kb from the domain of common insertion. Also depicted are structural motifs typical of MARs, including kinked DNA, curved DNA, AT-rich regions, ORI patterns and Topoisomerase II cleavage site patterns.

Previous studies showed that inoculation of neonatal mice with MoFe2 resulted in the development of T-cell lymphoma. Analysis of patterns of common proviral insertion in lymphomas revealed that MoFe2 utilized a set of CISs distinct from either parent virus from which it was constructed [39,40]. Sequence surrounding one of the previously described CISs in MoFe2-induced lymphomas, termed *MF8T *(*Rasgrp*1), was analyzed for the presence of MARs using a MAR-prediction program termed MAR-Finder . MAR-Finder is a statistical algorithm that analyzes the pattern density for characteristic DNA sequence motifs that predict the occurrence of MARs, including replication origins, TG-richness, curved DNA, kinked DNA, topoisomerase II recognition and cleavage sites and AT-richness. MAR-Finder has been previously validated for predicting the presence of MARs [34,41,42]. An alternative method to predict MARS is based on detecting the location and extent of stress-induced duplex destabilization (SIDD) through the use of a statistical algorithm termed WebSIDD [43-45]. Although this method has been validated to predict the presence of MARs accurately, recent evidence indicates that stress-induced destabilization of duplex DNA is not sufficient for a sequence to bind to the nuclear matrix; thus, the use of SIDD for the prediction of MARs may lead to false positives [46]. Using MAR-Finder, the results indicated the presence of two MARs in the 60-kb sequence surrounding *MF8T*, located 5.1-kb and 3.6-kb from the domain of common insertion (Figure 1). The predicted elements were observed to be enriched in motifs characteristic of MARS, including kinked DNA, curved DNA, AT-rich regions, origin of replication patterns and vertebrate and *Drosophila *topoisomerase II consensus sequences [26,27]. The close proximity of two MARS to the *MF8T *CIS suggested that integration near MARS may represent a mechanism for retroviral target site selection. To evaluate this possibility, the distance from proviral integration to predicted MARs was analyzed for three different groups of retroviruses, specifically murine gammaretroviruses (MoFe2, SL3-3 MuLV, MLV) human deltaretrovirus (HTLV-1) and lentivirus (HIV-1). Sequence information on MoFe2 integrations was obtained from the CISs and other insertion sites identified previously from a large collection of MoFe2-induced tumors [40]. MoFe2 integration sites were also analyzed from acutely infected SC-1 cells. In total, 42 MoFe2 integration sites were identified and analyzed in the present study. SL3-3 MuLV (SL3-3) integration sites had been previously identified from T-cell lymphomas in NIH-Swiss mice by inverse PCR [47]. In total, 86 SL3-3 integration sites were examined in the present study [47]. MLV and HIV-1 integration sites had been previously identified from HeLa cells infected with pseudotyped retroviral genomes [19]. From the 903 MLV and 379 HIV-1 insertions identified in that study, 49 (MLV) or 41 (HIV-1) integration sites for each virus were chosen at random for the present analysis. HTLV-1 integration sites from tumor-derived cells lines or from ATLL patients had been previously identified [8,12,34], 26 of which were examined in the present study. For each integration site examined in the present study, host-virus junction fragment sequences were obtained from GenBank  or the Mouse Retroviral Tagged Cancer Gene Database (RTCGD; ) and the integration sites were thereby positioned in the respective mouse or human genome using the NCBI mouse or human genome database  or .

**Figure 2:**
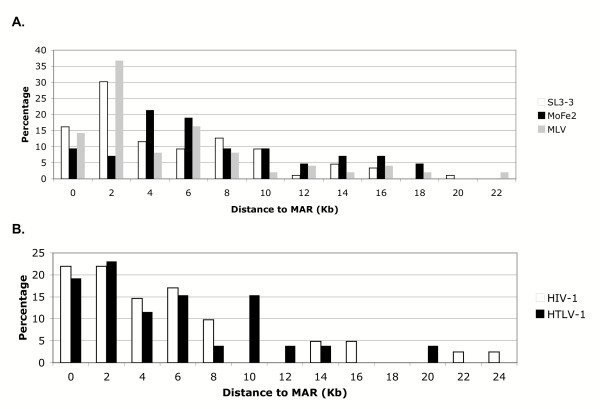
Distance of closest predicted MAR to proviral insertion site. Results are plotted as the percentage of integration events that occurred within 25-kb from a MAR using MAR-Finder for (A) gammaretroviruses (SL3-3, MoFe2 and MLV) and (B) HIV-1 and HTLV-1. SL3-3 and MLV integration distribution was significantly different than MoFe2 as determined by a one-way ANOVA followed by Tukey's multiple comparison test.

Initial analysis of insertion sites and their proximity to MARs revealed that some integrations were located more than 20-kb from a predicted MAR; therefore, to ensure a thorough identification of MARS in the vicinity of proviral integrations, 60-kb of sequence information surrounding each insertion site was obtained from the respective genome for analysis. Using 60-kb of sequence information surrounding each integration event, the distance from the proviral insertion site to the closest predicted MAR was plotted as the percentage of integration events analyzed (Figure 2). For the murine gammaretroviruses, the results indicated a preference to integrate within 2-kb of a predicted MAR. For example, 46% of SL3-3 integrations and 50% of MLV integrations occurred within 2-kb of a predicted MAR (Figure 2A). It has been reported that MARs occur every 10-kb in the mammalian genome [34,41]. Based on this report, a Monte Carlo simulation was performed where the mean distance to the closest MAR was computed under the assumption that viral integration occurs randomly with respect to regions that are predicted MARs and that MARs occur every 10-kb. The results indicated that, under these assumptions, the mean distance to the closest MAR during a random integration event would be 4-kb [34]. Thus, preferential integration near MARs is indicated for SL3-3 and MLV. By comparison, MoFe2 integration did not show the same preference (Figure 2A); rather, the distribution of MoFe2 integration sites in relation to MARs was significantly different from the distribution observed for SL3-3 and MLV (p < 0.01). In fact, the distribution of MoFe2 insertions in relation to MARs was consistent with the expectation for random integration. The same analysis was then performed on HTLV-1 and HIV-1 to determine if integration near MARs is also common for retroviruses that do not act in disease induction by insertional activation. The results indicated a preference for integration near MARs, since 43.9% of HIV-1 integrations and 42.3% of HTLV-1 integrations occurred within 2-kb of a predicted MAR (Figure 2B). As expected, a small percentage of integration events occurred more than 10-kb from a predicted MAR (Figure 2). In fact, for some integrations sites, the closest MAR in one direction was more than 60-kb away (data not shown). These results illustrate that, although MARs are predicted to be positioned at 10-kb intervals, there are regions of DNA that are either enriched or deficient in MARs as well.

**Figure 3:**
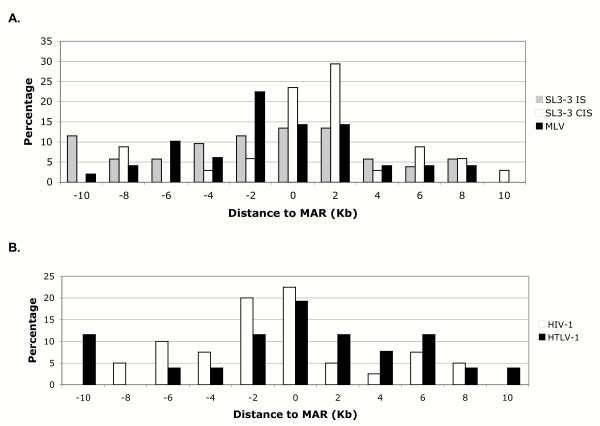
Position of MAR closest to the proviral integration site. The closest predicted MAR to the site of proviral insertion was determined to be located upstream or downstream from the site of insertion with respect to the transcriptional direction of the genetic locus. The results are plotted as the percentage of integrations that occurred up to 10-kb from a MAR for (A) SL3-3 insertions at single insertion site (SL3-3 IS), SL3-3 insertions at common insertion sites (SL3-3 CIS), MLV and (B) HTLV-1 and HIV-1.

Previous reports have indicated that MAR-mediated enhancement of viral gene expression is directional [32,34]. Other reports, in contrast, have indicated that MARs function to enhance gene expression in an orientation- and position- independent manner when located near the promoter [48]. To examine whether the preferred gammaretroviral integration near MARs is directional, it was next determined whether the closest predicted MAR was located upstream or downstream of the proviral integration site with respect to the transcriptional direction of the genetic locus. Results of the analysis, plotted as a percentage of integration events, indicated that the majority of MLV integrations occurred 1- to 2-kb from a predicted MAR on the downstream side (Figure 3A). For SL3-3, it was useful to consider independently the integrations previously identified as CISs in tumor DNA, since those integrations presumably function to activate nearby oncogenes [47]. Interestingly, SL3-3 insertions identified as CISs were found to integrate commonly within 2-kb from a predicted MAR and to be positioned on the upstream side. Of 31 such insertions examined, 29% were integrated within 2-kb upstream as compared to 5.8% integrated within 2-kb downstream of a predicted MAR (Figure 3A). By comparison, 44 SL3-3 integrations identified as only single insertion sites (ISs) did not show the same directional preference for integration near MARs (Figure 3A). These findings imply that SL3-3 integration immediately upstream of MARs within CISs may be related to insertional activation of the adjacent oncogene.

When examined by the same approach, analysis of HIV-1 and HTLV-1 integrations indicated that the majority of proviral insertions occurred near MARs, and 80% of the HIV-1 proviral integrations that occurred within 1- to 2-kb of a MAR were positioned downstream (Figure 3B). HTLV-1, while integrated preferentially within 2-kb of a MAR, did not show a position preference. A recent study also analyzed HIV-1 integration sites for their proximity to MARs. Consistent with our findings, that study indicated HIV-1 integration near MARs, specifically in the downstream position [35]. Another study, however, reported that MARs are commonly found downstream from the sites of HTLV-1 integrations [34]. As noted, we did not observe a position preference for HTLV-1 integrations relative to MARs (Figure 3B). The conflicting results may be due to the small sample size (n = 3) examined in the previous study.

**Figure 4:**
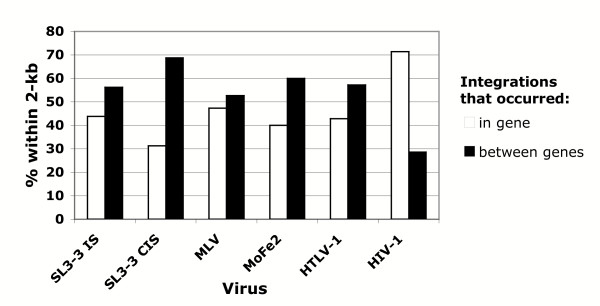
Analysis of the relationship between integration near a MAR and integration within or between genes. The percentage of integrations that occurred within 2-kb of a MAR is reported for those that occurred within a gene or between genes. Data are reported for SL3-3 insertions at single insertion site (SL3-3 IS), SL3-3 insertions at common insertion sites (SL3-3 CIS), MLV, MoFe2, HTLV-1 and HIV-1.

Several recent studies have reported that HIV-1, MLV and HTLV-1 integrate preferentially into genes [12,19,20]. With these findings in mind, SL3-3 and MoFe2 insertion sites were analyzed to determine whether a preference is evident for integration into RefSeqs. The analysis revealed that 17.6% SL3-3 integrations at CISs, 40.3% of SL3-3 integrations at single insertion sites, and 33.3% MoFe2 insertions occurred within RefSeqs (data not shown). By comparison, the frequency of integration into genes by random chance has been estimated at 22% [12,19,20]. Thus, preferential integration into genes was identified for MoFe2 and SL3-3 at single insertion sites, although not for SL3-3 integrated at CISs. Analysis was then performed to determine if preferred integration into genes was associated with integration near MARs. Using the NCBI mouse or human genome database, integration events were first grouped as to whether they occurred within or between genes. For each of the groups, the percentage of integrations that occurred within 2-kb of a predicted MAR was then determined (Figure 4). The results indicated no relationship to the nearest MAR when integration occurred within genes for SL3-3 at single insertion sites, MLV, MoFe2 or HTLV-1. In contrast, 71.4% of HIV-1 integrations that occurred within genes were observed to occur within 2-kb of a MAR. A strong relationship to MARs was also observed for SL3-3 integrations at CISs that occurred between genes. Of these integrations, 68.8% were observed to occur within 2-kb of a predicted MAR.

## Conclusion

Evidence is accumulating to indicate that proviral integration is not random, and that the secondary structure of DNA plays a major role in integration site selection [2-18]. In the present study, the integration patterns of three different groups of retroviruses with distinct mechanisms of disease induction were analyzed to determine if integration near MARs is a common mechanism of retroviral integration site selection. The results indicated that gammaretroviruses (MLV and SL3-3), lentivirus (HIV-1) and deltaretrovirus (HTLV-1) integrate preferentially near MARs, specifically within 2-kb (Figure 2). These results suggest that integration near MARs is a common mechanism of retroviral integration site selection. The findings are consistent with the previous identification of preferred integration sites that contained sequence motifs such as DNaseI hypersensitive sites [3-7], AT-rich regions [8], transcriptionally active regions [2,9-12], and regions of DNA bending, specifically regions with the most DNA distortion [14-18], all of which are motifs shared by MARs. A recent study analyzed the proximity of retroviral integration to MARs when the virus was delivered to the cell by infection or by electroporation of naked DNA [49]. The results showed a strong correlation for integration near MARs during infection, but not when transfected as naked DNA. These results further support a role for MARs in integration site selection during retroviral infection. There are several possible explanations for preferential integration near MARs. One possibility is that MARs, due to their position at the bases of chromatin loops, are likely to be the first region of the DNA encountered by the provirus when entering the nucleus. A second possibility is that MARs may represent the most accessible regions for integration in the DNA due to the open confirmation and high propensity for base-unpairing associated with the AT-richness. A third possibility relates to the observation that retroviruses may contain their own MARs. In fact, the mouse mammary tumor virus (MMTV) has been shown to contain a MAR in the LTR that binds a well characterized MAR-binding protein, SATB1 [50]. As the proviral pre-integration complex enters the nucleus, MAR binding proteins may bind and direct integration due to their affinity for binding to cellular MARs. It is known that sequence insertion within or near a MAR results in greatly reduced binding to the nuclear matrix [45]. In contrast, it has been shown that when retroviral integration occurs near MARs, contact with the nuclear matrix is maintained, suggesting that the presence of a MAR in the viral genome may stabilize the contact between the chromosomal MAR and the nuclear matrix [49].

The selective advantage of integration near MARs may be that it positions the provirus in close proximity to transcription, RNA processing and transport machinery that is localized at the nuclear matrix (reviewed in [25]), thus activating expression from the viral promoter. In addition, our findings suggest the possibility that integration near MARs may have a role in malignant induction, specifically by gammaretroviruses. SL3-3 proviruses integrated at CISs in tumor DNA were shown to position preferentially within 2-kb upstream from a MAR, whereas SL3-3 proviruses integrated at single insertion sites in the same tumors did not show the same preference (Figure 3). Considering that gammaretroviruses like SL3-3 induce malignancy through insertional activation of oncogenes at CISs, this observation suggests that SL3-3 integration immediately upstream of MARs may be associated with activation of adjacent cellular gene expression. Such an effect might occur by disruption of the normal function of the MAR, thus altering local chromatin conformation. Changes in chromatin conformation, leading to changes in gene expression, are known to contribute to malignancy (reviewed in [51]). Alternatively, integration at a specific distance and orientation with respect to a MAR may result in stimulation of expression from the viral promoter, thus enhancing virus-mediated activation of an adjacent cellular oncogene. Integration near MARs has also been implicated in malignant induction by small DNA tumor viruses [34]. These viruses do not induce disease by insertional activation; thus, the advantage of integration near MARs may relate to increased expression from the viral promoter.

Previous studies have reported that HIV-1, MLV, ASV and HTLV-1 prefer to integrate into genes [11,12,19,20]. In the present study, integration patterns of SL3-3 and MoFe2 were examined to determine if they also preferentially integrate into RefSeqs. Consistent with previous reports, our results indicated that SL3-3 proviruses at single insertion sites (40.3%) and MoFe2 proviruses (33.3%) integrate preferentially within RefSeqs as compared to the predicted frequency for random integrations (22%). SL3-3 proviruses integrated at CISs did not demonstrate the same preference, an observation consistent with the role of these integrants in enhancer-mediated activation of an adjacent oncogene. Of SL3-3 integrations at CISs that occurred between genes, 68.8% were observed within 2-kb of a predicted MAR (Figure 4). Taken together, these studies provide additional evidence that proviral integration is not random, that MARs influence retroviral integration site selection, and that integration near MARs may have a role in the insertional activation of oncogenes by gammaretroviruses. Understanding the pressures that influence retroviral integration site selection is critical for further knowledge of the mechanisms of retroviral pathogenesis and for the development of retroviral vectors for gene-therapy.

## Methods

### Isolation of MoFe2-MuLV host-virus junction fragments

MoFe2 proviral integrations were analyzed from lymphomas induced in a previous study [40] and from acutely infected tissue culture cells. For that purpose, 5 × 10^5 ^SC-1 murine fibroblasts at 25% confluence were infected with 10^5 ^infectious units (TCID_50_) of MoFe2 in the presence of 8 μg/ml of polybrene for 5 hours. Medium was removed, replaced with fresh EMEM with 10% FBS, and cells were harvested three days later. Genomic DNA was digested with DraI (TTT/AAA) or StuI (AGG/CCT), and libraries were constructed using Universal Genome Walker Kit (BD Biosciences) as described by the manufacturer. Libraries were constructed from both restriction enzyme digests to avoid introducing a bias for AT- or GC-rich sequences. Host-virus junction sequences were amplified by PCR using oligonucleotide primers and Universal Genome Walker Kit reagents as previously described [40]. Amplification products were cloned into TOPO-TA vector (Invitrogen Corp.) and submitted for automated sequence analysis. The resulting sequences were considered to represent valid MoFe2 integrations if they contained the viral 3' LTR and if the immediately flanking host sequence had a ≥95% identity to a single genomic locus.

### MAR analysis

A MAR prediction program termed MAR-Finder  was used to predict MARs on 60-kb intervals surrounding the insertion site using default detection and clipping parameters for SL3-3 (n = 86), MoFe2 (n = 42), MLV (n = 49), HIV-1 (n = 41) and HTLV-1 (n = 26) [34,41,42]. High scoring regions were considered valid if the average strength of a single peak representing a predicted MAR was >0.65 [34].

## Competing interests

The author(s) declare that they have no competing interests.

## Authors' contributions

CNJ performed all experimental and computer-based analyses. LSL directed the experimental design, implementation and interpretation of data. Both authors read and approved the final manuscript.
